# P-8. Assessment of 13-valent pneumococcal conjugate vaccine effectiveness among people living with HIV in the United States

**DOI:** 10.1093/ofid/ofae631.219

**Published:** 2025-01-29

**Authors:** Amanda C Miles, Sarah J Willis, Erica Chilson, Lindsay Grant, Christian Theilacker, Cassandra Hall-Murray, Qi Yan, Jeffrey T Vietri, Tamuno Alfred, Alejandro D Cane, Bradford D Gessner

**Affiliations:** Pfizer, New York, New York; Pfizer, New York, New York; Pfizer, New York, New York; Pfizer Inc., New York, New York; Pfizer Inc., New York, New York; Pfizer, Inc., Collegeville, Pennsylvania; Pfizer Inc., New York, New York; Pfizer, Inc., Collegeville, Pennsylvania; Pfizer, New York, New York; Pfizer, New York, New York; Pfizer Biopharma Group, Collegeville, Pennsylvania

## Abstract

**Background:**

People living with HIV (PLWH) have increased risk of pneumococcal infections. Although pneumococcal conjugate vaccines (PCVs) have been recommended for immunocompromised (IC) people, data regarding PCV effectiveness among PLWH are limited.

**Table 1.**
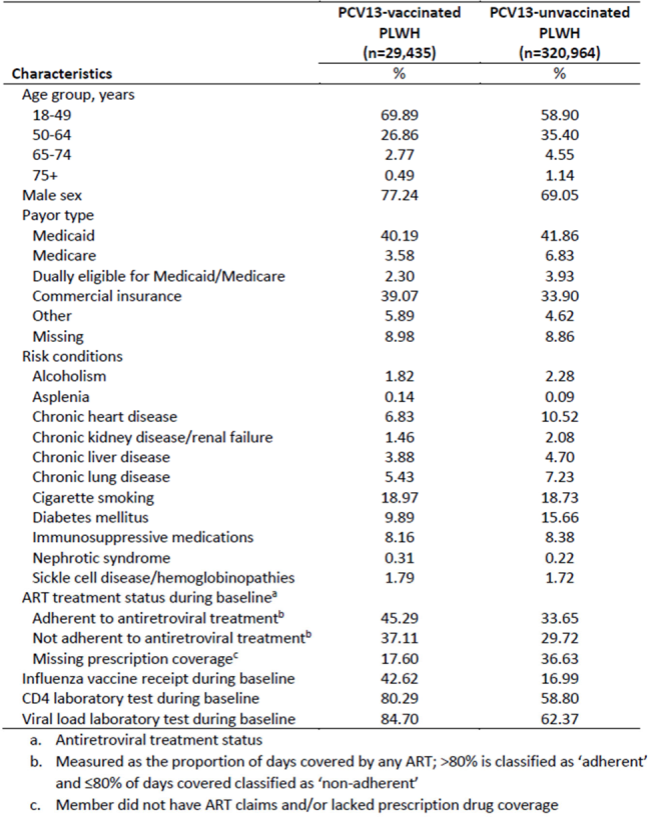
Baseline characteristics prior to weighting of 350,399 PLWH by receipt of PCV13 by end of baseline, United States, 2014-2022

**Methods:**

People with an HIV diagnosis code (index) from 1/1/2014 through 12/31/2021 were identified in US administrative claims. PLWH age ≥ 18 years without prior PCV13 vaccination were followed from six months post-index (baseline period) through 9/30/2022 for first episodes of invasive pneumococcal disease (IPD), pneumococcal pneumonia (PP) due to any serotype, and all-cause pneumonia (ACP) using diagnosis codes. Marginal structural Cox models estimated hazard ratios (HR) for each outcome and inverse probability weights were used to control for confounding/dropout. Vaccine effectiveness (VE) was estimated as (1-HR) x 100%. Candidiasis, all-cause diarrhea, and accidental injury served as negative control outcomes to assess residual confounding.

**Table 2.**
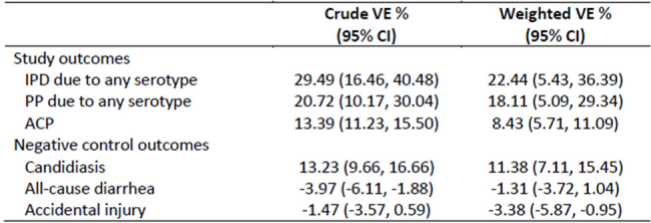
PCV13 VE against IPD, PP, ACP, and negative control outcomes among 350,399 PLWH, United States, 2014 - 2022

**Results:**

350,399 PLWH were included; 8% had received PCV13 during baseline, rising to 24% PCV13 by end of follow-up, and 25% received PPSV23. Mean follow-up was 2.9 years. Compared with PCV13-unvaccinated PLWH, greater proportions of PCV13-vaccinated PLWH were adherent to HIV medications and received CD4 tests, viral load tests, and influenza vaccination during baseline (Table 1). Overall weighted VEs (95% confidence intervals [CIs]) for IPD, PP, and ACP were 22% (5%, 36%), 17% (4%, 29%), and 8% (6%, 11%), respectively (Table 2). VE was highest for years 0 to < 3 of follow-up (0 to < 3 yr VE: 35% [13%, 51%]) for IPD, 26% [9%, 39%] for PP, and 11% [8%, 15%] for ACP) (Table 3). The negative control outcomes suggested residual confounding (Tables 2-3).

**Table 3.**
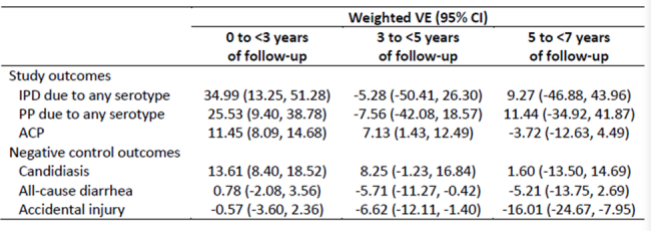
Weighted PCV13 VE against IPD, PP, ACP, and negative control outcomes among PLWH during 0 to < 3, 3 to < 5, and 5 to < 7 years of follow-up, United States, 2014 - 2022

**Conclusion:**

This study demonstrated real-world PCV13 VE against IPD and pneumonia among PLWH during the first three years of follow-up, but little evidence of VE thereafter. However, assessing VE after three years of follow-up was limited by decreased sample size and events. Overall, VE was relatively high considering pneumococcal outcomes were not specific to vaccine serotypes and ACP was not specific to pneumococcus. Residual confounding may also exist, but its direction remains unclear.

**Disclosures:**

**Amanda C. Miles, MPH**, Pfizer: Pfizer employee|Pfizer: Pfizer employee|Pfizer: Stocks/Bonds (Public Company)|Pfizer: Stocks/Bonds (Public Company) **Sarah J. Willis, PhD, MPH**, Pfizer, Inc.: Employment|Pfizer, Inc.: Stocks/Bonds (Private Company) **Erica Chilson, PharmD**, Pfizer Inc: Employee|Pfizer Inc: Stocks/Bonds (Public Company) **Lindsay Grant, PhD, MPH**, Pfizer, Inc: Employee of company|Pfizer, Inc: Stocks/Bonds (Private Company) **Christian Theilacker, MD, DTM&H**, Pfizer Inc: I am a Pfizer Employee|Pfizer Inc: Stocks/Bonds (Public Company) **Cassandra Hall-Murray, PharmD**, Pfizer, Inc.: employee|Pfizer, Inc.: Stocks/Bonds (Public Company) **Qi Yan, PhD, MS**, Pfizer: Pfizer employee|Pfizer: Stocks/Bonds (Public Company) **Jeffrey T. Vietri, PhD**, Pfizer Inc.: Employment|Pfizer Inc.: Stocks/Bonds (Public Company) **Tamuno Alfred, PhD**, Astellas Pharma: Astellas Pharma employee|Pfizer: Previous Pfizer employee|Pfizer: Stocks/Bonds (Public Company) **Alejandro D. Cane, MD, PhD**, Pfizer: Employee|Pfizer: Stocks/Bonds (Public Company)|Pfizer: Stocks/Bonds (Public Company) **Bradford D. Gessner, M.D., M.P.H.**, Pfizer: Employee|Pfizer: Stocks/Bonds (Public Company)

